# MicroRNA-223-3p Protect Against Radiation-Induced Cardiac Toxicity by Alleviating Myocardial Oxidative Stress and Programmed Cell Death *via* Targeting the AMPK Pathway

**DOI:** 10.3389/fcell.2021.801661

**Published:** 2022-01-17

**Authors:** Dao-ming Zhang, Jun-jian Deng, Yao-gui Wu, Tian Tang, Lin Xiong, Yong-fa Zheng, Xi-ming Xu

**Affiliations:** ^1^ Cancer Center, Renmin Hospital of Wuhan University, Wuhan, China; ^2^ Department of Pathology, Renmin Hospital of Wuhan University, Wuhan, China

**Keywords:** RIHD, MiR-223-3p, apoptosis, oxidative stress, AMPK

## Abstract

**Objectives:** Radiotherapy improves the survival rate of cancer patients, yet it also involves some inevitable complications. Radiation-induced heart disease (RIHD) is one of the most serious complications, especially the radiotherapy of thoracic tumors, which is characterized by cardiac oxidative stress disorder and programmed cell death. At present, there is no effective treatment strategy for RIHD; in addition, it cannot be reversed when it progresses. This study aims to explore the role and potential mechanism of microRNA-223-3p (miR-223-3p) in RIHD.

**Methods:** Mice were injected with miR-223-3p mimic, inhibitor, or their respective controls in the tail vein and received a single dose of 20 Gy whole-heart irradiation (WHI) for 16 weeks after 3 days to construct a RIHD mouse model. To inhibit adenosine monophosphate activated protein kinase (AMPK) or phosphodiesterase 4D (PDE4D), compound C (CompC) and AAV9-shPDE4D were used.

**Results:** WHI treatment significantly inhibited the expression of miR-223-3p in the hearts; furthermore, the levels of miR-223-3p decreased in a radiation time-dependent manner. miR-223-3p mimic significantly relieved, while miR-223-3p inhibitor aggravated apoptosis, oxidative damage, and cardiac dysfunction in RIHD mice. In addition, we found that miR-223-3p mimic improves WHI-induced myocardial injury by activating AMPK and that the inhibition of AMPK by CompC completely blocks these protective effects of miR-223-3p mimic. Further studies found that miR-223-3p lowers the protein levels of PDE4D and inhibiting PDE4D by AAV9-shPDE4D blocks the WHI-induced myocardial injury mediated by miR-223-3p inhibitor.

**Conclusion:** miR-223-3p ameliorates WHI-induced RIHD through anti-oxidant and anti-programmed cell death mechanisms *via* activating AMPK by PDE4D regulation. miR-223-3p mimic exhibits potential value in the treatment of RIHD.

## Introduction

With the increasing cancer incidence, radiotherapy (RT) has become an important treatment approach. On the one hand, RT improves the survival rate of cancer patients, but on the other, it also involves some inevitable complications of RT (Wang et al., 2019). Radiation-induced heart disease (RIHD) is one of the most serious complications, which is characterized by oxidative stress and cell loss (Ping et al., 2020). Mediastinal radiation therapy stimulates the atherosclerosis process, resulting in early onset coronary artery disease. Valvular disease caused by RT usually affects the left valve, with aortic regurgitation being the most common. In rare cases, it can lead to aortic stenosis that requires surgical intervention. Pericardial involvement includes acute and chronic pericardial disease and pericardial effusion (Raghunathan et al., 2017). A number of large clinical studies have confirmed that radiation therapy increases the risk of heart disease-related deaths (Laugaard Lorenzen et al., 2020). There is no clear treatment program to avoid or effectively eradicate the onset and subsequent development of RIHD. It is currently believed that RIHD is the result of the interaction of multiple mechanisms through multiple perturbed pathways; however, oxidative stress is considered to be the main one of them (Ping et al., 2020).

MicroRNAs (miRNAs) have gradually become the hot spot of RIHD research (Sarkozy et al., 2019). It has been considered that miRNAs regulate the signaling pathways and thus play a key role in the development of RIHD (Hawkins et al., 2019). miRNA is a type of single-stranded non-coding RNA with a length of approximately 19–25 nucleotides, which acts as a negative regulator of gene expression by binding to the 3′-untranslated region (UTR) of the target mRNA (Gomes et al., 2020). Emerging studies have shown that miRNAs participate in the regulation of the development of RIHD by influencing inflammation and oxidative damage (O'Connell et al., 2012; Bodega et al., 2019). A number of studies have shown that miR-223-3p is expressed in macrophages, platelets, hepatocytes, and cardiomyocytes and other cells (Zhang MW. et al., 2020). By targeting a variety of genes to regulate its cell activity, it is involved in the pathological progress of a variety of cardiovascular diseases (Zhang MW. et al., 2020). Chen et al. demonstrated that miR-223-3p inhibits the activation of inflammasomes by regulating the polarization of dendritic cells and then reduces the injury of autoimmune myocarditis (Chen et al., 2020). A recent study from Wu and colleagues showed that the expression level of serum miR-223-3p is closely related to the prognostic efficacy of patients with coronary heart disease (Wu et al., 2021). In addition, Han et al. demonstrated that miR-223-3p can inhibit atherosclerotic vascular calcification by regulating IL-6/STAT3 signal-mediated vascular smooth muscle cell (VSMC) transdifferentiation (Han et al., 2021). The results from Qin et al. showed that miR-223-3p can inhibit cardiac necrosis induced by myocardial ischemia-reperfusion. Adenosine monophosphate activated protein kinase (AMPK) is a key signal molecule that regulates energy metabolism, cell fate, and oxidative stress signal transduction in cardiomyocytes (Sun et al., 2006). Recent studies have shown that activating AMPK alleviated oxidative stress and apoptosis (Ichihara et al., 2006). Furthermore, miR-223-3p exhibited an indispensable role in regulating apoptosis and redox homeostasis in a variety of diseases (Matsuzaki and Ochiya, 2018; Tang et al., 2018; Zhang and Cheng, 2021). In this study, we aim to explore the role and potential mechanism of miR-223-3p in the progression of RIHD.

## Materials and Methods

### Material

Enzyme-linked immunosorbent assay (ELISA) assay including lactate dehydrogenase (LDH), creatine kinase MB isoenzyme (CK-MB), were purchased from MyBioSource (CA, United States). PKA (protein kinase A) colorimetric activity kit. Dihydrodichlorofluorescein diacetate (DCF-DA) and caspase-3 assay kit were obtained from ThermoFisher Scientific (CA, United States). Total antioxidant capacity (TAOC) assay kits, total superoxide dismutase (SOD) activity assay kits, malondialdehyde (MDA) assay kits, 3-nitrotyrosine (3-NT) assay kits, and 8-hydroxy 2 deoxy guanosine (8-OHdG) assay kits were purchased from Abcam (Cambridge, United Kingdom). ELISA^PLUS^ kit was purchased from Roche Applied Science (Mannheim, Germany). Caspase 3/7 activity using a kit from Promega (WI, United States). Total collagen assay kit and soluble collagen assay kit were purchased from Abcam (Cambridge, United Kingdom). Phosphorylated (P−) AMPK, total (T−) AMPK, antibodies, and β-actin were purchased from Cell Signaling Technology (MA, United States). p53, cAMP, PKA, NRF2, HO-1, α-SMA, fibronectin, and phosphodiesterase 4D (PDE4D) were purchased from Abcam (Cambridge, United Kingdom). Compound C was obtained from Selleck Chemicals (Texas, United States). An AAV9 system harboring shPDE5D, shRNA, miR-223-3p mimic, miR-223-3p mimic inhibitor, or negative control was generated by HanBio Technology (Shanghai, China).

### Animals

C57BL/6 mice (6–8 weeks) were purchased from Chinese Academy of Medical Sciences and Peking Union Medical College. Mice received a single dose of 20 Gy whole-heart irradiation (WHI) or sham therapy under anesthesia using an X-RAD 160–225 radiator (CT, United States) with a 2 mm AI filter (Lee et al., 2012). To overexpress miR-223-3p, mice received 1 × 10^11^ viral genome of AAV9-miR-223-3p mimic (mimic) or AAV9-scramble (con) by tail vein injection according to a previous study. To investigate miR-223-3p deficiency in WHI model, mice received 1 × 10^11^ viral genome of AAV9-miR-223-3p inhibitor (inhibitor) or AAV9-negative control (NC) by tail vein injection. After the 4th week injection, mice were subjected to WHI. For AMPK inhibition, compound C (20 mg/kg) was intraperitoneally injected every 2 days at 1 week before WHI stimulation (Zhang X. et al., 2020). For PDE4D inhibition, mice received 1 × 10^11^ viral genome of AAV9-shPDE4D (shPDE4D) or AAV9-shRNA (shRNA) by tail vein injection at 4 weeks before WHI therapy (Ma et al., 2021). After 15 weeks WHI, the cardiac echocardiographic and hemodynamic measurements were performed, and then all mice were sacrificed with euthanasia by an overdose of sodium pentobarbital. The animal experiments were performed according to the Guide for the Care and Use of Laboratory Animals published by the US National Institutes of Health. The protocol was approved by the institutional animal care and use committee of Renmin Hospital of Wuhan University.

### Echocardiography and Hemodynamic

Serial transthoracic echocardiography was performed according to previously described method (Fan D. et al., 2017; Liu et al., 2019). For pressure–volume loops, a pressure probe catheter (TX, United States) was inserted into the left carotid artery and then retrogradely advanced into the left ventricle to record the hemodynamic parameters.

### ELISA Assay

The DNA damage biomarkers, apoptosis biomarkers, and oxidation/antioxidation biomarker level in serum or cardiac from each group were detected by utilizing ELISA kits according to the manufacturer's protocol.

### Collagen Assay

Total collagen content was determined by measuring hydroxyproline levels in the left ventricles as previously described. In order to evaluate the content of soluble collagen, mouse heart tissues of each group were placed in pepsin solution, and the supernatant was precipitated with trichloroacetic acid. The precipitate was hydrolyzed with HCL, and the hydroxyproline level was detected by the commercial kit. The total collagen content minus the amount of soluble collagen equals the insoluble collagen content.

### Cardiac Injury Biomarkers Measurement

To detect plasma LDH and CK-MB levels, blood samples were collected from mice. CK-MB and LDH were detected to reflect the cardaic injury according to standard procedures.

### Real-Time PCR

We used TRIzol reagent to isolate total mRNA. We used the SmartSpec Plus Spectrophotometer (Bio-Rad) to detect mRNA purity with OD260/OD280 ratios. A total of 2 μg of mRNA was reverse transcribed into cDNA with cDNA Synthesis Kit (Roche Diagnostics). Gene expression analysis was carried out using the Fast SYBR Green master mix (Applied Biosystems) and the QuantStudio 12k Flex real-time PCR (RT-PCR) system (Switzerland). Results were normalized to β-actin mRNA as an internal control. miRNA level was determined using Bulge-Loop miRNA qRT-PCR Starter Kit (Guangzhou, China). U6 was used as the internal control of miRNA.

### Western Blotting

Western blotting was performed according to previously described method (Guo et al., 2020). Protein samples were prepared from heart tissues. Whole-cell lysates or tissue lysates were prepared in radioimmunoprecipitation assay (RIPA) buffer supplemented with 1 mmol/L phenylmethanesulfonyl fluoride (PMSF) and 1% protease inhibitor cocktail before use. For Western blotting analysis, the lysates were clarified, denatured, separated on 10% polyacrylamide gels by sodium dodecyl sulfate polyacrylamide gel electrophoresis (SDS-PAGE) and transferred to polyvinylidene difluoride (PVDF) membranes. Detection of the target proteins on membranes was performed using standard techniques. The abundance 1 of the proteins was determined by densitometry. Raw data of band intensity were normalized to a control protein such as β-actin.

### Data and Statistical Analysis

All experiments were randomized and blinded. Data are presented as means ± standard deviation. Statistical analysis was performed with GraphPad Prism version 8.0.1 and the SPSS 18.0 software package (IL, United States). Multiple comparisons were carried out using Tukey's test following significant one-way or two-way ANOVA. Unpaired *t*-test was to compare significance between the two groups. *p*-values <0.05 were considered to be statistically significant.

## Results

### miR-223-3p Mimic Reduces Myocardial Damage and Dysfunction in RIHD Mice

**FIGURE 1:**
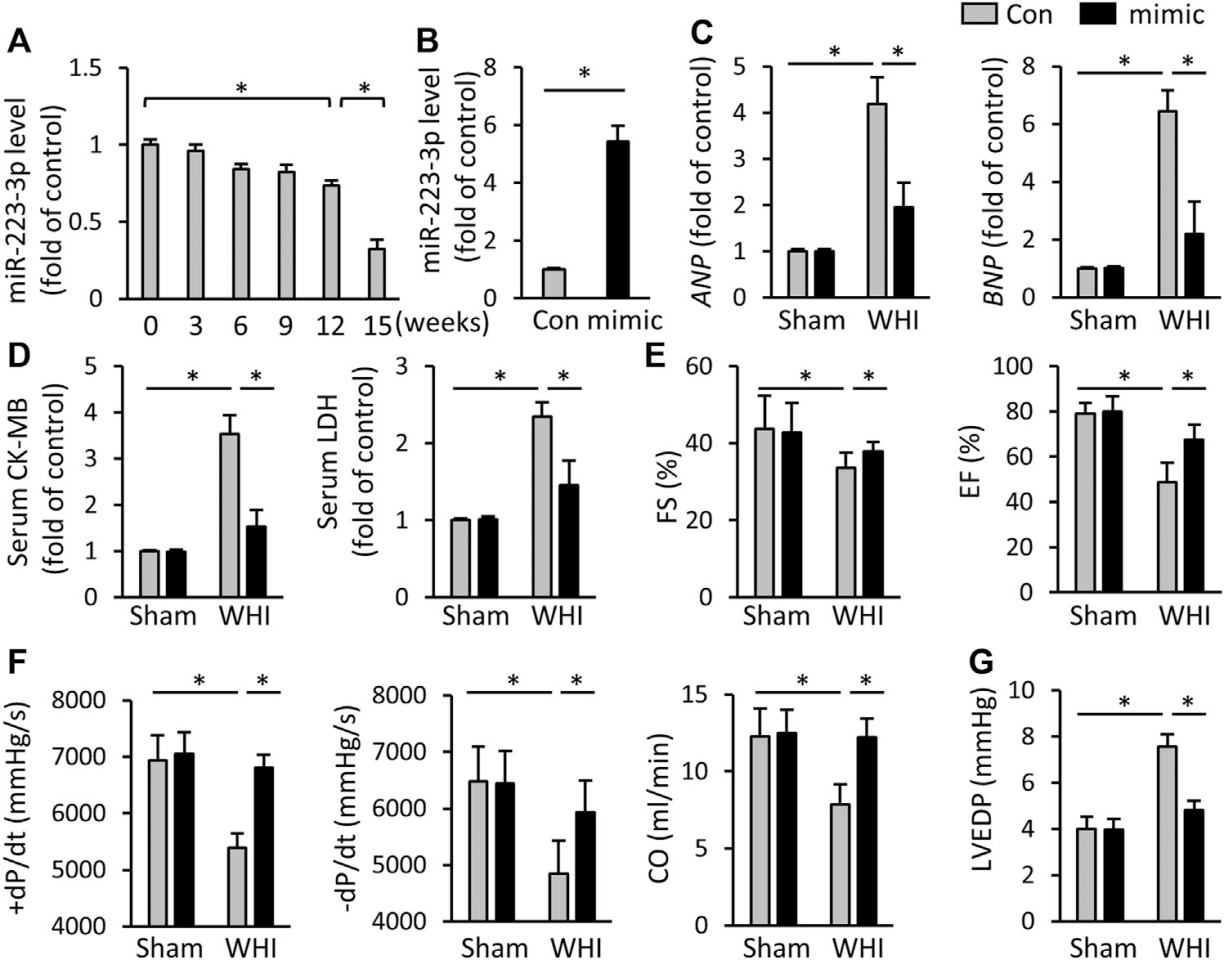
miR-223-3p mimic attenuates cardiac injury and dysfunction. **(A)** Relative level of miR-223-3p in cardiac with 20 Gy WHI were detected at the indicated times (*n* = 6). **(B)** Relative level of miR-223-3p in mice hearts with or without miR-223-3p mimic treatment (*n* = 5). **(C)** Relative mRNA levels of *ANP* and *BNP* in the hearts (*n* = 6). **(D)** Serum biomarkers related to cardiac injury with or without miR-30d-5p mimic treatment upon WHI stimulation, including CK-MB and LDH (*n* = 6). **(E**–**G)** Echocardiographic and hemodynamic parameters were detected at 16 weeks after WHI or sham therapy (*n* = 6). CO, cardiac output; All data are presented as means ± SD. *p < 0.05 vs. the matched group.

As shown in Figure 1A, WHI treatment significantly reduced the expression of miR-223-3p in mice hearts in a radiation-time dependent manner. At the 15th week after WHI stimulation, the level of miR-223-3p reached the lowest. To clarify the role of miR-223-3p, mice were injected with adenovirus harboring miR-223-3p mimic, the efficiency of which is shown in Figure 1B. After 15 weeks of WHI therapy, the levels of heart failure biomarkers *ANP* and *BNP* were increased, while miR-223-3p mimic alleviated this alternation (Figure 1C). The serum CK-MB and LDH, indicators associated with cardiac injury, were reduced with miR-223-3p mimic treatment (Figure 1D). Then, we examined the cardiac function and found that miR-223-3p mimic ameliorated WHI-induced cardiac dysfunction by improving the left fractional shortening (FS), left ventricular ejection fraction (EF), maximal rate of the left ventricular pressure fall or increase (±dP/dt), and cardiac output (CO) (Figures 1E, F). In addition, the end-diastolic pressure in the left ventricle (LVEDP) of WHI mice was restored with miR-223-3p mimic treatment (Figure 1G). These data indicate that miR-223-3p mimic alleviates cardiac injury and dysfunction in mice with WHI stimulation.

### miR-223-3p Mimic Inhibits Oxidative Stress Damage and Programmed Cell Death

**FIGURE 2:**
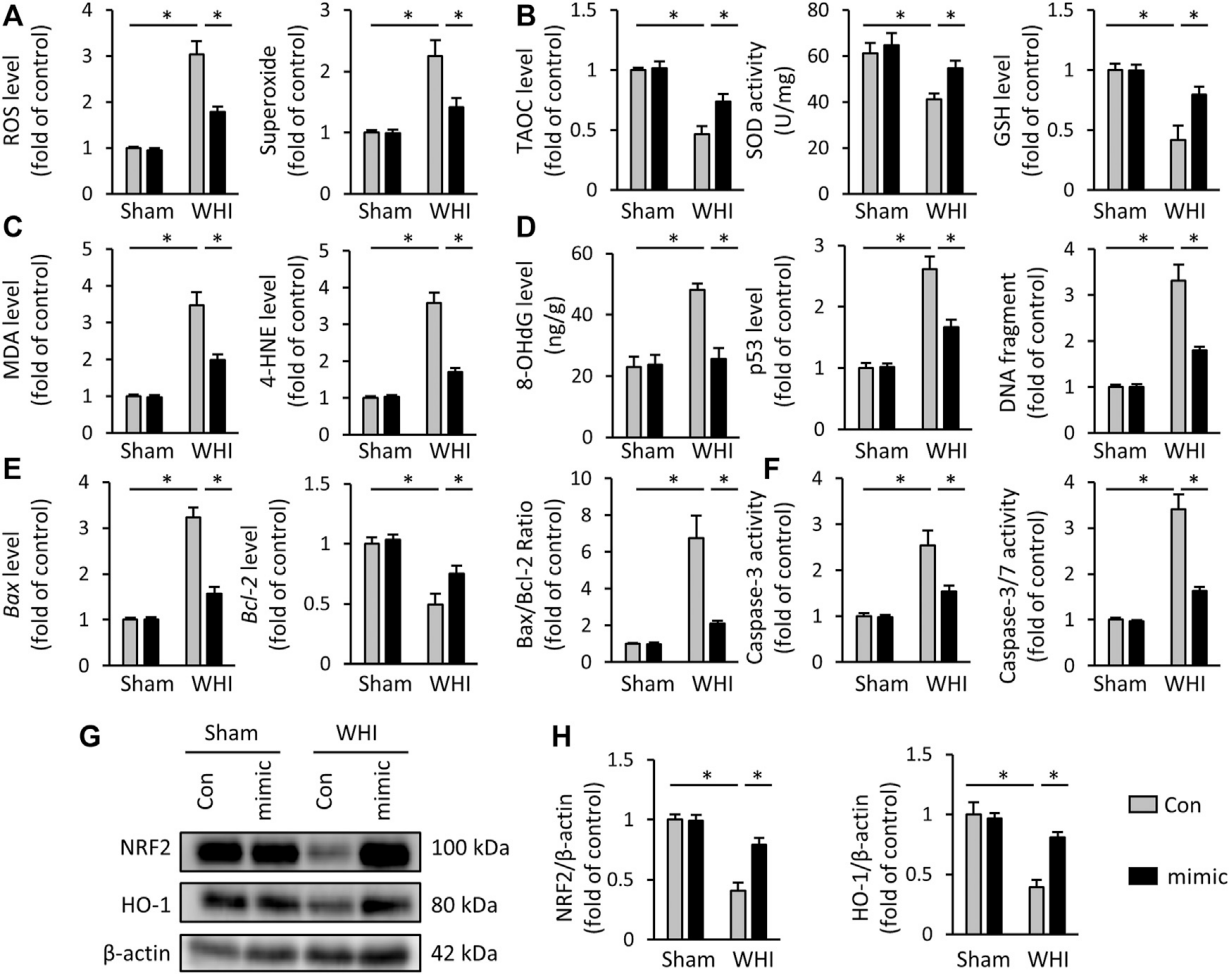
miR-223-3p mimic inhibits oxidative stress and programmed cell death in WHI mice. **(A)** ROS and superoxide level in the hearts with or without miR-223-3p mimic (*n* = 6). **(B)** TAOC and total SOD activity and GSH level detected by ELISA assay (*n* = 6). **(C)** Oxidative products levels in mice hearts (*n* = 6). **(D)** Quantitative results of 8-OHdG, p53, and DNA fragment in each group (*n* = 6). **(E)** Relative mRNA levels of *Bax* and *Bcl-2* and the ratio of Bax/Bcl-2 in the hearts (*n* = 6). **(F)** Quantitative results of caspase-3 and caspase-3/7 activity in each group (*n* = 6). **(G**, **H)** Relative protein expression of NRF2 and HO-1 in each group (*n* = 6). All data are presented as means ± SD. *p < 0.05 vs. the matched group.

Due to the critical role of oxidative stress and programmed cell death in the progression of RIHD, we assessed the effect of miR-223-3p mimic on redox reaction and cell loss in the hearts of RIHD mice. As shown in Figures 2A, B, WHI increased the accumulation of reactive oxygen species (ROS) and superoxide but decreased in miR-223-3p mimic-treated mice. The increase of peroxides needs to be swept by antioxidants, such as SOD and glutathione (GSH). We tested the TAOC and the levels of SOD and GSH and found that miR-223-3p mimic also reduced WHI-related SOD and GSH consumption and increased the TAOC (Figure 2B). In addition, increased production of 4-HNE and MDA were found in WHI mice, indicating increased oxidative stress, which were also alleviated with miR-223-3p mimic treatment (Figure 2C). Radiation, both acute and chronic exposure, induces oxidative stress, causing oxidative damage and DNA damage. Cellular DNA damage includes single strand breaks and double strand breaks in cellular DNA and also 8-OHdG in cellular DNA. Therefore, by detecting DNA biomarkers in myocardial tissue, we detected that DNA biomarkers, such as 8-OHdG, p53, and DNA fragments, were elevated in the myocardial tissue of WHI mice and reduced by miR-223-3p (Figure 2D). RT-PCR results showed that the hearts of WHI mice exhibited higher programmed cell death compared with WHI mice treated with miR-223-3p mimic, as reflected by levels of *Bax* and *Bcl-2* and the ratio of Bax/Bcl-2 (Figure 2E). Consistent with the RT-PCR results, the levels of Caspase-3 and Caspase3/7, as programmed cell death executive molecules, were decreased in miR-223-3p mimic-treated mice, detected by ELISA (Figure 2F). It has been reported that NRF2 acts as a redox-sensitive transcription factor, which inhibits oxidative damage in RIHD (Fan Z. et al., 2017; Cao et al., 2021). As shown in Figures 2G, H, WHI significantly reduced the protein levels of NRF2 and its downstream HO-1 in mice hearts, which was rescued by miR-223-3p mimic treatment. Therefore, we conclude that miR-223-3p mimic inhibits oxidative stress damage and programmed cell death in radiated hearts.

### miR-223-3p Mimic Reduces Myocardial Fibrosis in RIHD Mice

**FIGURE 3:**
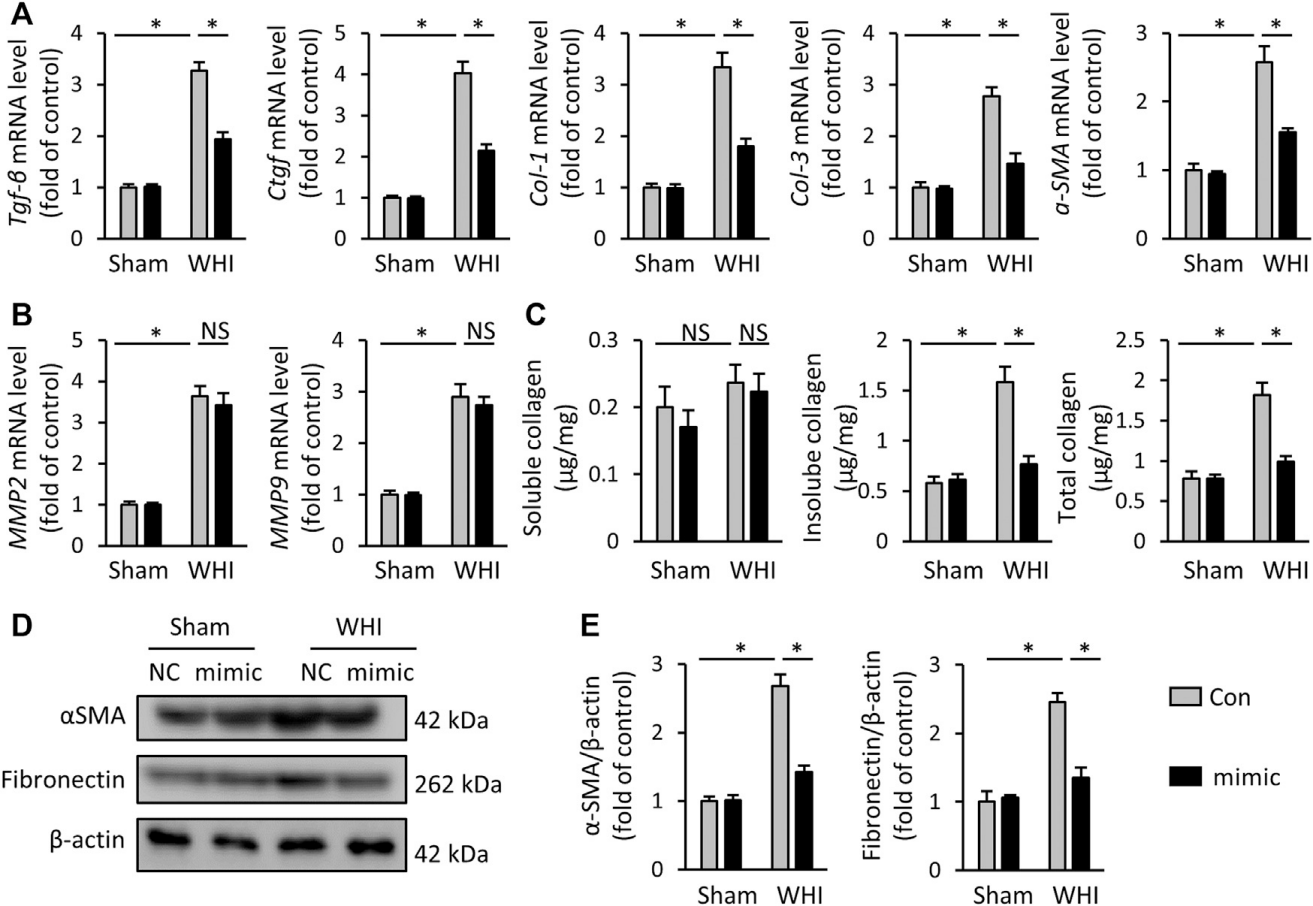
miR-223-3p mimic reduces myocardial fibrosis in RIHD mice. **(A)** Relative mRNA levels of *Tgf-β*, *Ctgf*, *Col-1*, *Col-3*, and *α-SMA* in the hearts (*n* = 6). **(B)** Relative mRNA levels of *MMP2* and *MMP9* in each group (*n* = 6). **(C)** Total, soluble, and insoluble collagen content were assessed in each group (*n* = 6). **(D**, **E)** Relative protein expression of α-SMA and fibronectin in each group (*n* = 6). All data are presented as means ± SD. *p < 0.05 vs. the matched group.

Radiation-induced myocardial fibrosis is a chronic stage of RIHD, which is the main cause of cardiac stiffness and cardiac dysfunction (Ma et al., 2019; Sarkozy et al., 2019). Next, we assessed the role of miR-223-3p mimic on the transcription of key molecules related to myocardial fibrosis, such as *TGF-β*, *Ctgf*, *col-1*, *col-3*, and *α-SMA*. As shown in Figure 3A, after the mice received 15 weeks WHI, the mRNA elevation of the pro-fibrosis markers induced by WHI was alleviated by miR-223-3p mimic administration. MMP2 and MMP9 are both main components of the extracellular matrix, which are elevated in WHI mice (Figure 3B). Interestingly, MMP2 (WHI+Con vs. WHI+mimic, 3.63 vs. 3.40) and MMP9 (WHI+Con vs. WHI+mimic, 2.89 vs. 2.74) in WHI mice were unaffected by miR-223-3p mimic. ELISA data also showed that the total insoluble (mainly cross-linked) collagen content in the left ventricle of mice was decreased in miR-223-3p mimic treatment mice with WHI stimulation, without alteration of the soluble forms (Figure 3C). Western blot detection revealed that the elevation of α-SMA and fibronectin expression induced by WHI were alleviated by miR-223-3p mimic injection (Figures 3D, E). These data indicated that miR-223-3p mimic reduced myocardial fibrosis in RIHD mice.

### miR-223-3p Inhibitor Aggravates Oxidative Stress and Programmed Cell Death in RIHD Mice

**FIGURE 4:**
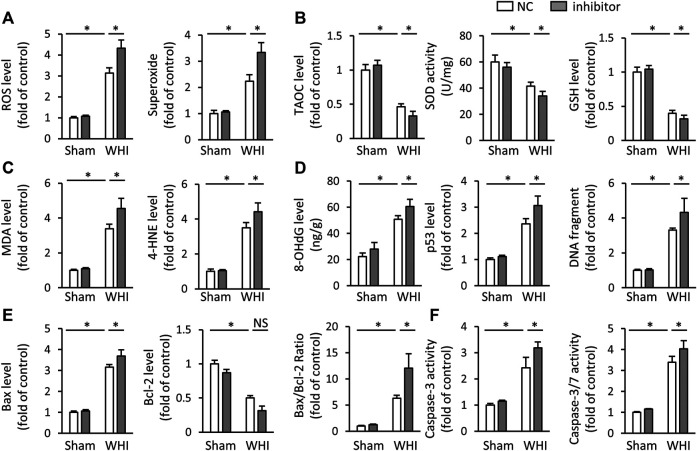
miR-223-3p inhibitor aggravates oxidative stress and programmed cell death in RIHD mice. **(A)** ROS and superoxide level in the hearts with or without miR-223-3p inhibitor (*n* = 6). **(B)** The relative levels of TAOC and total SOD activity and GSH (*n* = 6). **(C)** Oxidative products levels in mice with or without miR-223-3p inhibitor (*n* = 6). **(D)** Quantitative results of 8-OHdG, p53, and DNA fragment in each group (*n* = 6). **(E)** Relative mRNA levels of *Bax* and *Bcl-2* and the ratio of Bax/Bcl-2 in the hearts (*n* = 6). **(F)** Quantitative results of caspase-3 and caspase-3/7 activity in each group (*n* = 6). All data are presented as means ± SD. *p < 0.05 vs. the matched group.

We also used miR-223-3p inhibitor to determine whether inhibiting miR-223-3p would aggravate oxidative stress and programmed cell death in RIHD mice. As shown in Figures 4A–C, the miR-223-3p inhibitor significantly increases the accumulation of ROS and peroxide in the hearts after WHI therapy, with lower levels of the TAOC, SOD, and GSH, Correspondingly, the productions of MDA and 4-HNE were enhanced with miR-223-3p inhibitor injection. Correspondingly, miR-223-3p inhibitor administration increased the DNA damage, as determined by the levels of 8-OHdG, p53, and DNA fragments (Figure 4D). As expected, we found that miR-223-3p inhibitor significantly increased the level of pro-apoptotic marker Bax mRNA and reduced the anti-apoptotic Bcl-2 mRNA expression under WHI stimulation (Figure 4E). Accordingly, miR-223-3p inhibitor further promoted the increase of Caspase-3 and Caspase-3/7 levels in WHI mice. These data provide evidence that miR-223-3p inhibitor aggravate oxidative stress and programmed cell death induced by WHI therapy.

### miR-223-3p Inhibitor Aggravates Myocardial Damage and Dysfunction in RIHD Mice

**FIGURE 5:**
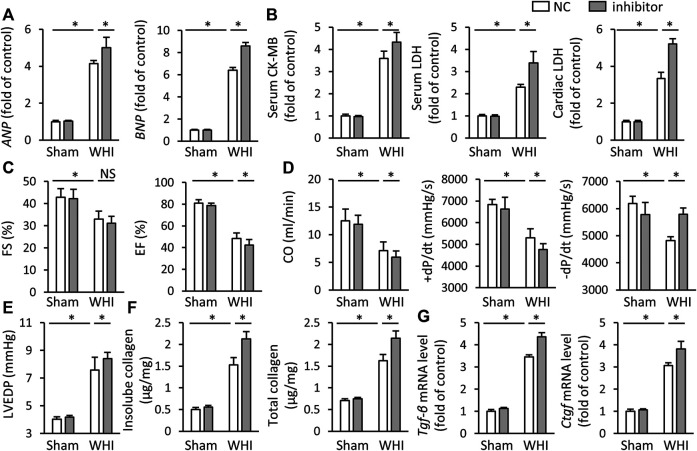
miR-223-3p inhibitor aggravates myocardial damage and dysfunction in RIHD mice. **(A)** Relative mRNA levels of *ANP* and *BNP* in the hearts (*n* = 6). **(B)** Serum and cardiac biomarkers related to cardiac injury with or without miR-30d-5p mimic treatment upon WHI stimulation, including CK-MB and LDH (*n* = 6). **(C**–**E)** Echocardiographic and hemodynamic parameters (*n* = 6). **(F)** Total and insoluble collagen content were assessed in each group (*n* = 6). **(G)** Relative mRNA levels of *Tgf-β* and *Ctgf* in the hearts (*n* = 6). All data are presented as means ± SD. *p < 0.05 vs. the matched group.

Because of miR-223-3p aggravating oxidative stress and programmed cell death in RIHD mice, we also investigated the effect of miR-223-3p inhibitor on cardiac injury and dysfunction induced by WHI. As shown in Figure 5A, compared with WHI mice, miR-223-3p inhibitor further increased the levels of *ANP* and *BNP* (Figure 5A). As expected, miR-223-3p inhibitor also promotes cardiac injury, as reflected by serum levels of CK-MB and LDH, as well as LDH levels in cardiac tissue (Figure 5B). The greater deterioration of EF and FS in the mice with pretreatment of miR-223-3p inhibitor compared with WHI+NC group was detected by echocardiographic examination (Figure 5C). Correspondingly, the hemodynamic analysis showed that CO, ±dP/dt, and LVEDP further worsened after miR-223-3p inhibitor injection (Figures 5D, E). Furthermore, mice pretreated with miR-223-3p inhibitor promoted the transcription of the pro-fibrotic gene (*TGF-β* and *CTGF*) with WHI stimulation and accordingly increased the deposition of total collagen and insoluble collagen in the left ventricle (Figures 5F, G). Besides, the levels of MMP2 and MMP9 were not significantly different between the WHI+NC group and WHI+inhibitor group, further indicating that miR-223-3p unaffected the MMP2 and MMP9 (data not shown). Therefore, we concluded that miR-223-3p inhibitor aggravates cardiac injury and dysfunction induced by WHI.

### miR-223-3p Mimic Improves WHI-Induced RIHD by Activating AMPK

**FIGURE 6:**
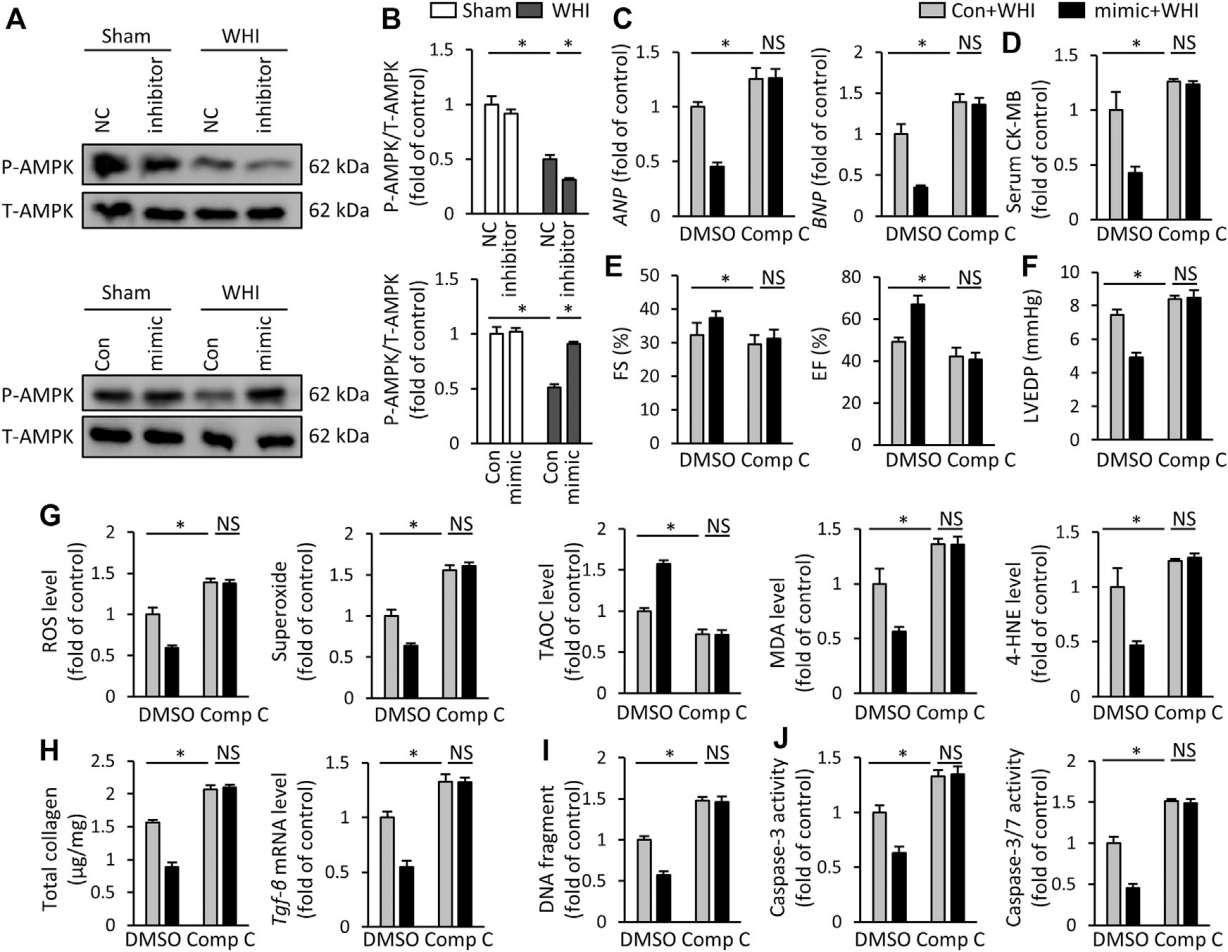
miR-223-3p mimic improves WHI-induced RIHD by activating AMPK **(A**, **B)** Protein expression of total AMPK and phosphorylation AMPK (*n* = 6). **(C)** Relative mRNA levels of *ANP* and *BNP* (*n* = 6). **(D)** Serum CK-MB level (*n* = 6). **(E**, **F)** The alteration in FS, EF, and LVEDP in mice with or without compound C administration (*n* = 6). **(G)** ROS content and oxidative products in mice (*n* = 6). **(H)** Determination of the total collagen content and relative mRNA level of TGF-β (*n* = 6). **(I)** Quantitative results of DNA fragments (*n* = 6). **(J)** Quantitative results of caspase-3 activity and caspase-3/7 activity (*n* = 6). All data are presented as means ± SD. *p < 0.05 vs. the matched group.

To further explore the mechanism of miR-223-3p in RIHD, we collected heart tissues from each group of mice. Western blot data showed that miR-223-3p mimic prevented the decrease of AMPK phosphorylation induced by WHI, while miR-223-3p inhibitor further aggravated the reduction of AMPK phosphorylation (Figures 6A, B). Next, the AMPK inhibitor CompC was used to block AMPK activity *in vivo* to verify whether the effect of miR-223-3p was dependent on AMPK activation. We found that miR-223-3p mimic-mediated cardiac protection was significantly eliminated by CompC administration, as shown by unchanged *ANP*, *BMP*, and serum CK-MB levels (Figures 6C, D). Consistent with unaltered cardiac injury markers, the protective effect of miR-223-3p mimic on RIHD was also blocked by AMPK inhibitors, as reflected by FS, EF, and LVEDP (Figures 6E, F). In addition, CompC also blocked the effect of miR-223-3p mimic on oxidative stress, as determined by ROS, superoxide, MDA, 4-HNE, and TAOC (Figure 6G). Correspondingly, miR-223-3p mimic failed to ameliorate myocardial fibrosis, DNA damage, and programmed cell death in WHI mice with CompC administration (Figures 6H–J). Hence, we consider that miR-223-3p exerted a cardioprotective role in WHI mice by activating AMPK.

### miR-223-3p Regulates PDE4D/AMPK Signaling

**FIGURE 7:**
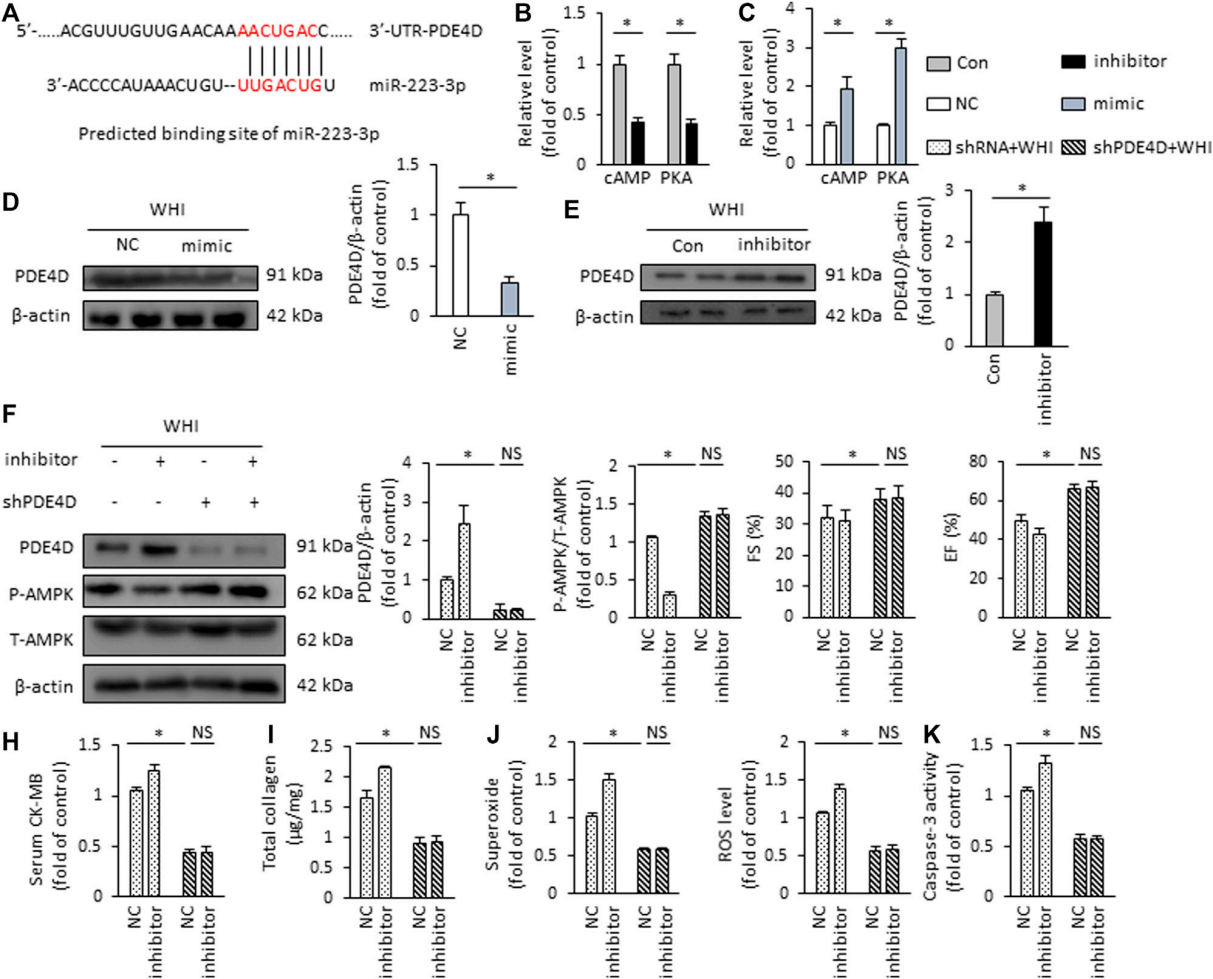
miR-223-3p regulates PDE4D/AMPK signaling **(A)** Putative binding site of miR-223-3p by TargetScan Mouse 7.1. **(B**, **C)** Relative cAMP level and PKA activity in the hearts (*n* = 6). **(D**, **E)** Protein expression of PDE4D detected by Western blot (*n* = 6). **(F)** The protein expression alteration of PDE4D and AMPK in the hearts (*n* = 6). **(G)** The alteration in FS and EF in mice (*n* = 6). **(H)** Serum CK-MB level detected by ELISA kit (*n* = 6). **(I)** Total collagen content in the mice hearts (*n* = 6). **(J)** ROS content in the hearts (*n* = 6). **(K)** Quantitative results of caspase-3 activity (*n* = 6). All data are presented as means ± SD. *p < 0.05 vs. the matched group.

Finally, we investigated the possible mechanism of AMPK activation regulation by miR-223-3p. In predicting the miR-223-3p regulatory target site by TargetScan Mouse 7.1, we revealed that the supposed banding site of miR-223-3p is in the 3′-UTR of PDE4D (Figure 7A). PDE4D is a specific phosphodiesterase (PDE) isoenzyme that regulates the hydrolysis of cAMP and the downstream PKA pathway (Yun et al., 2019). Therefore, the reduction of PDE4D downstream cAMP and PKA activity were found in the mice with miR-223-3p inhibitor treatment, while it is increased by miR-223-3p mimic (Figures 7B, C). In addition, miR-223-3p significantly inhibits the expression of PDE4D in WHI mice (Figures 7D, E). In order to verify whether miR-223-3p is involved in the regulation of AMPK activation *via* PDE4D, mice were injected with AAV9-shPDE4D into the tail vein to silence the expression of PDE4D in the mice hearts. As shown in Figure 7F, shPDE4D significantly inhibited the protein expression of PDE4D in myocardial tissue and rescued the inhibition of AMPK phosphorylation mediated by miR-223-3p inhibitor. Moreover, PDE4D deficiency restored the deterioration of cardiac dysfunction mediated by miR-223-3p inhibitor (Figure 7G), as suggested by EF and FS. miR-223-3p inhibitor-associated increased level of serum CK-MB was also blunted by shPDE4D injection (Figure 7H). Consistent with the cardiac function, AAV9-shPDE4D reduced the effects of miR-223-3p inhibitor in WHI mice, as suggested by the levels of total collagen, superoxide, ROS, and caspase-3 activity (Figures 7I–K). Collectively, we demonstrated that miR-223-3p suppressed the expression of PDE4D by directly binding to the 3′-UTR of PDE4D and subsequently activated AMPK to regulate the progression of RIHD.

## Discussion

With the increasing cancer incidence, RT has become an important treatment approach. Long-term radiation therapy, especially chest cancer radiation therapy, leads to RIHD. In this study, we aimed to explore the role and mechanism of miR-223-3p in RIHD. The main findings are as follows: 1) the expression of miR-223-3p in the hearts of WHI mice with a radiation time-dependent manner; 2) miR-223-3p exerted cardioprotective effects on WHI induced heart injury *via* reducing cardiac oxidative damage and programmed cell death; 3) miR-223-3p mimic ameliorates WHI-induced radiocardiotoxicity *via* AMPK activation; and 4) PDE4D is involved in AMPK activation by miR-223-3p. In conclusion, our results demonstrated for the first time that miR-223-3p showed potential value in RIHD treatment.

As we know, the heart is an organ with high oxygen consumption, and cardiomyocytes contain a large number of mitochondria. The ROS are mainly produced by the mitochondria in the heart (Pohjoismaki and Goffart, 2017). Therefore, it is important to explore the mechanism of ROS regulation in the progression of heart disease. When ROS are produced in large quantities, excess free radicals are neutralized by increasing the production of intracellular antioxidants (including GSH, SOD, etc.). However, along with the antioxidants consumed, the fracture of the balance between the oxidation and the antioxidant system leads to ROS accumulation, which causes cardiac injury (Boengler et al., 2017). Studies have shown that oxidative stress also plays an important role in RIHD (Cuomo et al., 2016). Myocardial tissue contains a large amount of water, and the onset of water decomposition during radiation exposure subsequently produces a large amount of ROS (Vorotnikova et al., 2010). When the production of oxygen-free radicals exceeds the antioxidant capacity of cardiomyocytes, DNA damage occurs. According to previous reports, there are many forms of DNA damage, which can significantly change the structure of DNA and ultimately lead to cell cycle arrest, cell death, mutation, and other effects (Marnett et al., 2003; Yamamori et al., 2012). The DNA damage repair (DDR) pathway is mediated by a variety of functional proteins and is an important mechanism for repairing DNA damage and ensuring the integrity of the genome (Martinet et al., 2002). The p53 gene is one of the main effectors of the DDR signaling pathway (Bhattacharya and Asaithamby, 2016). Analogously, the Bax family also alters with radiation stimulation, leading to increased apoptosis (Sridharan et al., 2014). In addition to DNA damage, ROS can also cause lipid and protein peroxidation and activate a variety of signaling pathways (Dent et al., 2003). Consistent with the results of previous studies, we found that the markers of myocardial injury in WHI mice were significantly increased, accompanied by cardiac dysfunction and hemodynamic alternation. However, cardiac injury and dysfunction was improved in WHI mice with miR-223-3p mimic pretreatment. In addition, we found that WHI increased the production of ROS, superoxide, MDA, and 4-HNE and reduced the antioxidant-related biomarkers, including TAOC, SOD, and GSH. However, miR-223-3p mimic alleviated the alternations of oxidative stress induced by WHI. NRF2 is an antioxidant transcription factor that regulates the expression of a variety of antioxidant enzymes (Tonelli et al., 2018) and is particularly important for maintaining the balance of the oxidation/antioxidant system in WHI mice. In this study, we observed that miR-223-3p mimic significantly upregulated the protein levels of NRF2 and its downstream HO-1 in WHI mice, which also partly accounts for the fact that miR-223-3p mimic alleviated oxidative stress level induced by WHI stimulation.

Apoptosis is a main form of myocardial programmed cell death that is regulated by the Bcl-2 family and caspase family of proteins (Brentnall et al., 2013; Shi et al., 2017). Numerous studies have shown that animal models with cardiac disease involve apoptosis (Baba et al., 2018; Bruni et al., 2018). Programmed cell death is a tightly regulated signaling process required for myocardial homeostasis. Intrinsic apoptosis, mediated by the mitochondrial outer membrane permeabilizatione, is mitochondrial-centered cell death. The initiation and execution of these processes are regulated by the Bcl-2 and caspase protein family (Danial and Korsmeyer, 2004; Galluzzi et al., 2012). The activation of Bcl-2 family member Bax results in the release of mitochondrial outer membrane permeabilization and cytochrome c from the interstitial space into the cytoplasm (Eskes et al., 2000; Wei et al., 2000; Wei et al., 2001). Subsequently, cytochrome c binds Apaf-1 to form apoptotic bodies and activate caspase-9. Once activated, caspase-9 directly cleaves and activates caspase-3 and caspase-7 (Li et al., 1997; Srinivasula et al., 1998). Hence, the level of caspase-3 and caspase-7 together with mRNA levels of Bcl-2 and Bax reflect the degree of programmed cell death. Our data suggested that miR-223-3p mimic ameliorated WHI-induced programmed cell death. The reduction of programmed cell death implied more viable cardiomyocytes involved in the pumping force of the heart. Replacement fibrosis replaces dead cardiomyocytes with extracellular matrix tissue and fibroblasts, preserving tissue integrity at the expense of muscle bundle continuity (Talman and Ruskoaho, 2016). The reduction of myocardial fibrosis improves the elevation of myocardial compliance, which partly accounts for the effects of miR-223-3p mimic in improving cardiac function. Consist with the result of cell death. The DNA damage and myocardial fibrosis were aggravated in mice with miR-223-3p inhibitor but alleviated with miR-223-3p mimic treatment. Overall, our data indicated that miR-223-3p prevents WHI-induced cardiac injury and dysfunction through anti-oxidant and anti-programmed cell death mechanisms.

Many studies have shown that AMPK, as the core protein of heart energy metabolism, plays a critical role in the regulation of oxidative stress (Lai et al., 2016). AMPK activating promotes Nrf2 nuclear transfer, and then Nrf2 enters the nucleus and combines with the HO-1 promoter to promote the transcription of antioxidants (Lv et al., 2017). Meanwhile, activated AMPK is also essential for the introduction of cytosolic fatty acids into mitochondria. Phosphorylation of AMPK inactivates acetyl-CoA carboxylase (ACC), resulting in a decrease in the concentration of malonyl-CoA (Qi and Young, 2015). Consistently, we found miR-223-3p mimic alleviated WHI-induced cardiac injury by activating AMPK and further verified that inhibition of AMPK by the CompC administration completely offset the anti-apoptotic and antioxidant effects of miR-223-3p mimic in WHI mice. According to TargetScan Mouse 7.1 software, we determined the putative binding site of miR-223-3p in PDE4D 3′-UTR. Western blot results verified that miR-223-3p inhibits the expression of PDE4D. PDE4D, as a hydrolase of cAMP, inhibits the activity of downstream PKA by reducing the concentration of cAMP. PKA is a key kinase activated by AMPK (Chen et al., 2019). Furthermore, PDE4D inhibition by AAV9-shPDE4D injection reversed the inhibitory effect of miR-223-3p inhibitor on AMPK phosphorylation. Consequently, the pro-inflammatory and pro-oxidant effects of miR-223-3p inhibitor in WHI mice were also alleviated with AAV9-shPDE4D injection.

However, there are still some limitations in the current research. First, why is miR-223-3p downregulated in WHI mice hearts? Secondly, it is uncertain whether miR-223-3p inhibits WHI-induced programmed cell death depending on the production of ROS. Third, whether there are other signal pathways involved in the regulation of miR-223-3p on RIHD is not known. Finally, does miR-223-3p inhibit the effect of RT on tumors? All these issues need to be explored in future research.

In conclusion, we found for the first time that miR-223-3p inhibits programmed cell death and oxidative stress at least in part by regulating AMPK activation to protect against RIHD. This study provides potential ideas for RIHD treatment.

## Data Availability

The original contributions presented in the study are included in the article/Supplementary Material; further inquiries can be directed to the corresponding authors.
